# 4-Hy­droxy-3-[(*E*)-3-phenyl­prop-2-eno­yl]-2*H*-chromen-2-one

**DOI:** 10.1107/S1600536811029801

**Published:** 2011-07-30

**Authors:** Afef Ghouili, Rached Ben Hassen

**Affiliations:** a Unité de chimie des Matériaux, ISSBAT, Université de Tunis-ElManar, 9 Avenue Dr Zoheir SAFI, 1006 Tunis, Tunisia

## Abstract

In the title mol­ecule, C_18_H_12_O_4_, the phenyl ring is twisted by 23.2 (1)° from the mean plane of the chromene system. In the crystal, weak inter­molecular C—H⋯O hydrogen bonds link mol­ecules into zigzag chains extending in the [010] direction. An intra­molecular O—H⋯O hydrogen bond is also present.

## Related literature

For related structures, see: Traven *et al.* (2000 ▶); Sun & Cui (2008 ▶); Mechi *et al.* (2009 ▶); Hamdi *et al.* (2010 ▶); Asad *et al.* (2010 ▶). For the synthesis of coumarin chalcones, see: Claisen & Claparede (1881 ▶).
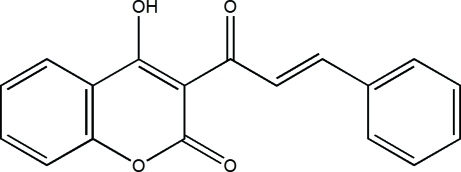


## Experimental

### 

#### Crystal data

C_18_H_12_O_4_
*M* *_r_* = 292.28
Monoclinic, 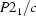

*a* = 11.8040 (5) Å
*b* = 3.8860 (5) Å
*c* = 29.7190 (5) Å
β = 97.164 (5)°
*V* = 1352.58 (18) Å^3^
*Z* = 4
Mo *K*α radiation
μ = 0.10 mm^−1^
*T* = 296 K
0.3 × 0.14 × 0.06 mm

#### Data collection

Bruker SMART CCD area-detector diffractometer
Absorption correction: numerical (*SADABS*; Bruker, 2003 ▶) *T*_min_ = 0.861, *T*_max_ = 0.865
11154 measured reflections
2983 independent reflections
1404 reflections with *I* > 2σ(*I*)
*R*_int_ = 0.070

#### Refinement

*R*[*F* ^2^ > 2σ(*F* ^2^)] = 0.069
*wR*(*F* ^2^) = 0.300
*S* = 1.04
2983 reflections
203 parameters
H atoms treated by a mixture of independent and constrained refinement
Δρ_max_ = 0.54 e Å^−3^
Δρ_min_ = −0.69 e Å^−3^

### 

Data collection: *SMART* (Bruker, 2003 ▶); cell refinement: *SAINT* (Bruker, 2003 ▶); data reduction: *SAINT*; program(s) used to solve structure: *SHELXS97* (Sheldrick, 2008 ▶); program(s) used to refine structure: *SHELXL97* (Sheldrick, 2008 ▶); molecular graphics: *DIAMOND* (Brandenburg, 1999 ▶); software used to prepare material for publication: *WinGX* (Farrugia, 1999 ▶).

## Supplementary Material

Crystal structure: contains datablock(s) I, global. DOI: 10.1107/S1600536811029801/cv5125sup1.cif

Structure factors: contains datablock(s) I. DOI: 10.1107/S1600536811029801/cv5125Isup2.hkl

Supplementary material file. DOI: 10.1107/S1600536811029801/cv5125Isup3.cml

Additional supplementary materials:  crystallographic information; 3D view; checkCIF report

## Figures and Tables

**Table 1:** Hydrogen-bond geometry (Å, °)

*D*—H⋯*A*	*D*—H	H⋯*A*	*D*⋯*A*	*D*—H⋯*A*
C5—H5⋯O3^i^	0.93	2.57	3.350 (5)	142
O1—H2⋯O2	0.99 (7)	1.51 (7)	2.413 (4)	149 (6)

Symmetry code: (i) 
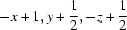
.

**Fig. 1.**
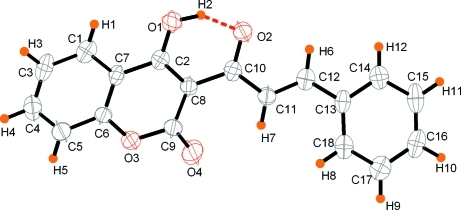
The molecular structure of (I) showing 50% probability displacement ellipsoids and the atomic numbering. Dashed line denotes hydrogen bond.

## supplementary crystallographic information

### Comment

In continuation of our structural and biological studies of coumarin derivatives (Mechi *et al.*, 2009; Hamdi *et al.*, 2010), we present the crystal structure of the title compound (I) - a new chalcone of the coumarin.

In (I) (Fig. 1), all bond lengths and angles are normal and correspond to those observed in related structures (Mechi *et al.*, 2009; Asad *et al.*, 2010). The presence of α, β-unsaturated ketone is indicated by the short O2–C10 and C11–C12 bond lengths of 1.289 (5)Å and 1.327 (5) Å, respectively, and the O2–C10–C11 and C10–C11–C12 bond angles of 118.2 (4) ° and 121.8 (4) °, respectively. The structure exhibits intramolecular hydrogen bonding between the hydroxyl oxygen and the ketonic oxygen in the coumarin group (Table 1). The chromen-2-one is twisted out of the plane of the phenyl ring (C13– C14) at 23.2 (1) °. The linkage between the coumarin system and phenyl ring is quite conjugated with bond lengths C10–C11 = 1.457 (5) Å, C11–C12 = 1.327 (5) Å, and C12–C13 = 1.440 (5) Å, suggesting that all non-hydrogen atoms between the electron-donors and acceptors are highly conjugated, leading to a π-bridge for the charge transfer from phenyl ring to coumarin system. Similar geometry has been observed in coumarin chalcone analogues (Mechi *et al.*, 2009; Sun & Cui, 2008). Consequently, the C10–O2 = 1.289 (5) Å is elongated as compared with its mean value found in 3-acetyl-4hydroxycoumarin (1.253 Å) (Traven *et al.*, 2000) owing to the localization of the hydroxyl hydrogen (H2) between the O2 ketonic oxygen and the hydroxyl oxygen O1. The O1–H2 distance (0.99 (7) Å) in (I) is shorter than that in related compounds - C_18_H_10_O_7_ (1.22 (7) Å) (Mechi *et al.*, 2009) and C_18_H_10_Cl_2_O_4_ (1.27 (2) Å) (Asad *et al.*, 2010).  It should be noted that the C9–O4 bond length (1.198 (5) Å) is less than that (1.210 Å) observed in 3-acetyl-4hydroxycoumarin (Traven *et al.*, 2000). It was concluded that it was a substantial difference for stabilizing the H atom of the hydroxyl group when we changed the nature of the substituted *R* group (from H to Cl and to OCH3).

In the crystal structure, weak intermolecular C– H···O hydrogen bonds (Table 1) link molecules into zigzag chains extended in [010].

### Experimental

The new chalcone (I) was synthesized using the Claisen Schmidt reaction (Claisen & Claparede, 1881), by the condensation of 3-acetyl-4hydroxycoumarin (1g, 4.9 mmol) and aromatic benzaldehyde (6.4 mmol, 0.5 ml) in chloroform (5 ml) in the presence of one drop of piperidine. The mixture was refluxed in a water bath for 2 h. After cooling at room temperature, a yellow solid was obtained in good yield, filtered, washed with ethanol, and dried in air. Yellow block-shaped single crystals of the title compound, suitable for X-ray structure determination, were recrystalized by slow evaporation of dichloromethane (CH_2_Cl_2_)  at room temperature after several days. Yield: 1.1 g (80%). mp= 499K. IR: ν 3468 (OH), 1690(*s*) (>C=O), 1578 (C= C), 1272(*s*) (*sym*) (C-O-C); 1HNMR: δppm: 7.4-8.1 (m, 10H, Ar-H+ Hethyl), 16.1(*s*,1H,OH). 13C NMR (ppm): 192.6(CO); 181.56 (C2); 160.22 (C9); 100.8 (C8), 116.32-147.368 (C arom); 124.39 (Cethyl1), 154.79 (Cethyl2),

### Refinement

H2 atom was located on a difference map and refined isotropically. The remaining H atoms were positioned geometrically (C–H 0.93 Å) and refined using a riding model, with U_iso_(H) = 1.2 U_eq_(C).

### Crystal data

**Table:**

C_18_H_12_O_4_	*F*(000) = 608
*M**_r_* = 292.28	*D*_x_ = 1.435 Mg m^−^^3^
Monoclinic, *P*2_1_/*c*	Melting point: 489 K
Hall symbol: -P2ybc	Mo *K*α radiation, λ = 0.71073 Å
*a* = 11.8040 (5) Å	Cell parameters from 203 reflections
*b* = 3.8860 (5) Å	µ = 0.10 mm^−^^1^
*c* = 29.7190 (5) Å	*T* = 296 K
β = 97.164 (5)°	Plate, yellow
*V* = 1352.58 (18) Å^3^	0.3 × 0.14 × 0.06 mm
*Z* = 4	

### Data collection

**Table:**

Bruker SMART CCD area-detector diffractometer	2983 independent reflections
Radiation source: fine-focus sealed tube	1404 reflections with *I* > 2σ(*I*)
graphite	*R*_int_ = 0.070
φ and ω scans	θ_max_ = 27.2°, θ_min_ = 1.4°
Absorption correction: numerical (*SADABS*; Bruker, 2003)	*h* = −15→14
*T*_min_ = 0.861, *T*_max_ = 0.865	*k* = −4→4
11154 measured reflections	*l* = −38→37

### Refinement

**Table:**

Refinement on *F*^2^	Primary atom site location: structure-invariant direct methods
Least-squares matrix: full	Secondary atom site location: difference Fourier map
*R*[*F*^2^ > 2σ(*F*^2^)] = 0.069	Hydrogen site location: inferred from neighbouring sites
*wR*(*F*^2^) = 0.300	H atoms treated by a mixture of independent and constrained refinement
*S* = 1.04	*w* = 1/[σ^2^(*F*_o_^2^) + (0.1616*P*)^2^] where *P* = (*F*_o_^2^ + 2*F*_c_^2^)/3
2983 reflections	(Δ/σ)_max_ < 0.001
203 parameters	Δρ_max_ = 0.54 e Å^−^^3^
0 restraints	Δρ_min_ = −0.69 e Å^−^^3^

### Special details

**Table:**

Geometry. All e.s.d.'s (except the e.s.d. in the dihedral angle between two l.s. planes) are estimated using the full covariance matrix. The cell e.s.d.'s are taken into account individually in the estimation of e.s.d.'s in distances, angles and torsion angles; correlations between e.s.d.'s in cell parameters are only used when they are defined by crystal symmetry. An approximate (isotropic) treatment of cell e.s.d.'s is used for estimating e.s.d.'s involving l.s. planes.
Refinement. Refinement of *F*^2^ against ALL reflections. The weighted *R*-factor *wR* and goodness of fit *S* are based on *F*^2^, conventional *R*-factors *R* are based on *F*, with *F* set to zero for negative *F*^2^. The threshold expression of *F*^2^ > σ(*F*^2^) is used only for calculating *R*-factors(gt) *etc*. and is not relevant to the choice of reflections for refinement. *R*-factors based on *F*^2^ are statistically about twice as large as those based on *F*, and *R*- factors based on ALL data will be even larger.

### Fractional atomic coordinates and isotropic or equivalent isotropic displacement parameters (Å^2^)

**Table:**

	*x*	*y*	*z*	*U*_iso_*/*U*_eq_	
O1	0.0403 (2)	0.2994 (9)	0.14926 (11)	0.0562 (9)	
O2	0.0632 (2)	0.0415 (9)	0.07711 (9)	0.0559 (9)	
C1	0.1298 (4)	0.5245 (10)	0.23674 (14)	0.0449 (11)	
H1	0.0526	0.5667	0.2281	0.054*	
C2	0.1481 (3)	0.2566 (10)	0.16162 (13)	0.0372 (9)	
C10	0.1719 (3)	0.0080 (10)	0.08756 (13)	0.0403 (10)	
C3	0.1795 (4)	0.6154 (11)	0.27952 (14)	0.0531 (12)	
H3	0.1357	0.7162	0.2999	0.064*	
C14	0.1811 (4)	−0.5082 (11)	−0.06124 (13)	0.0473 (11)	
H12	0.1031	−0.5413	−0.0610	0.057*	
C12	0.1891 (4)	−0.2364 (10)	0.01330 (13)	0.0418 (10)	
H6	0.1098	−0.2428	0.0093	0.050*	
C7	0.1950 (3)	0.3694 (9)	0.20651 (12)	0.0362 (9)	
C8	0.2206 (3)	0.1046 (10)	0.13289 (12)	0.0363 (9)	
C15	0.2328 (4)	−0.6146 (11)	−0.09835 (14)	0.0546 (13)	
H11	0.1895	−0.7214	−0.1227	0.065*	
C11	0.2385 (4)	−0.1217 (10)	0.05304 (13)	0.0421 (10)	
H7	0.3177	−0.1241	0.0590	0.050*	
C4	0.2948 (4)	0.5566 (11)	0.29212 (14)	0.0532 (12)	
H4	0.3282	0.6208	0.3209	0.064*	
C6	0.3095 (3)	0.3138 (10)	0.22020 (12)	0.0379 (9)	
C9	0.3401 (4)	0.0457 (11)	0.14931 (13)	0.0445 (10)	
C16	0.3483 (4)	−0.5624 (11)	−0.09925 (14)	0.0536 (12)	
H10	0.3824	−0.6308	−0.1244	0.064*	
C5	0.3595 (4)	0.4068 (11)	0.26304 (14)	0.0507 (11)	
H5	0.4368	0.3669	0.2718	0.061*	
O3	0.3791 (2)	0.1608 (8)	0.19242 (9)	0.0485 (8)	
C17	0.4132 (4)	−0.4082 (12)	−0.06275 (15)	0.0538 (12)	
H9	0.4912	−0.3759	−0.0630	0.065*	
O4	0.4098 (3)	−0.0973 (11)	0.12993 (11)	0.0753 (12)	
C18	0.3610 (4)	−0.3021 (11)	−0.02576 (13)	0.0471 (11)	
H8	0.4046	−0.1954	−0.0015	0.057*	
C13	0.2453 (4)	−0.3519 (10)	−0.02429 (13)	0.0386 (9)	
H2	0.022 (6)	0.181 (17)	0.120 (2)	0.13 (2)*	

### Atomic displacement parameters (Å^2^)

**Table:**

	*U*^11^	*U*^22^	*U*^33^	*U*^12^	*U*^13^	*U*^23^
O1	0.0409 (18)	0.085 (2)	0.0422 (18)	0.0068 (16)	0.0031 (14)	−0.0133 (17)
O2	0.0428 (18)	0.083 (2)	0.0404 (17)	0.0022 (16)	−0.0005 (14)	−0.0144 (16)
C1	0.056 (3)	0.040 (2)	0.041 (2)	0.0013 (19)	0.017 (2)	0.0005 (18)
C2	0.040 (2)	0.038 (2)	0.034 (2)	−0.0007 (17)	0.0080 (17)	0.0014 (17)
C10	0.045 (2)	0.040 (2)	0.037 (2)	−0.0008 (18)	0.0070 (19)	0.0017 (18)
C3	0.086 (4)	0.044 (2)	0.034 (2)	−0.002 (2)	0.021 (2)	−0.0042 (18)
C14	0.053 (3)	0.049 (2)	0.038 (2)	0.005 (2)	0.001 (2)	0.0004 (19)
C12	0.048 (2)	0.044 (2)	0.034 (2)	0.0010 (19)	0.0072 (19)	−0.0007 (18)
C7	0.045 (2)	0.036 (2)	0.028 (2)	−0.0032 (17)	0.0103 (17)	0.0047 (16)
C8	0.042 (2)	0.038 (2)	0.029 (2)	−0.0014 (17)	0.0038 (17)	0.0021 (16)
C15	0.084 (4)	0.047 (3)	0.030 (2)	0.011 (2)	0.001 (2)	−0.0057 (19)
C11	0.046 (2)	0.047 (2)	0.033 (2)	0.0025 (19)	0.0059 (18)	−0.0005 (18)
C4	0.076 (3)	0.050 (3)	0.032 (2)	−0.010 (2)	0.000 (2)	−0.0010 (19)
C6	0.043 (2)	0.041 (2)	0.030 (2)	−0.0036 (18)	0.0080 (17)	0.0012 (17)
C9	0.043 (2)	0.056 (3)	0.034 (2)	0.008 (2)	0.0060 (19)	−0.0003 (19)
C16	0.078 (4)	0.052 (3)	0.034 (2)	0.018 (2)	0.016 (2)	0.003 (2)
C5	0.055 (3)	0.055 (3)	0.041 (2)	−0.009 (2)	−0.002 (2)	0.000 (2)
O3	0.0369 (16)	0.072 (2)	0.0357 (16)	0.0048 (15)	0.0014 (12)	−0.0042 (14)
C17	0.060 (3)	0.056 (3)	0.047 (3)	0.007 (2)	0.014 (2)	0.003 (2)
O4	0.053 (2)	0.123 (3)	0.050 (2)	0.032 (2)	0.0068 (17)	−0.021 (2)
C18	0.060 (3)	0.047 (2)	0.033 (2)	0.001 (2)	0.005 (2)	0.0004 (18)
C13	0.052 (2)	0.035 (2)	0.029 (2)	0.0043 (18)	0.0049 (18)	0.0029 (16)

### Geometric parameters (Å, °)

**Table:**

O1—C2	1.290 (5)	C7—C6	1.379 (5)
O1—H2	0.99 (7)	C8—C9	1.452 (5)
O2—C10	1.289 (5)	C15—C16	1.383 (7)
C1—C3	1.378 (6)	C15—H11	0.9300
C1—C7	1.391 (5)	C11—H7	0.9300
C1—H1	0.9300	C4—C5	1.354 (6)
C2—C8	1.411 (5)	C4—H4	0.9300
C2—C7	1.447 (5)	C6—O3	1.371 (4)
C10—C8	1.447 (5)	C6—C5	1.383 (5)
C10—C11	1.457 (5)	C9—O4	1.198 (5)
C3—C4	1.385 (7)	C9—O3	1.381 (5)
C3—H3	0.9300	C16—C17	1.384 (6)
C14—C15	1.388 (6)	C16—H10	0.9300
C14—C13	1.394 (6)	C5—H5	0.9300
C14—H12	0.9300	C17—C18	1.388 (6)
C12—C11	1.327 (5)	C17—H9	0.9300
C12—C13	1.440 (5)	C18—C13	1.385 (6)
C12—H6	0.9300	C18—H8	0.9300
			
C2—O1—H2	107 (4)	C12—C11—C10	121.8 (4)
C3—C1—C7	120.0 (4)	C12—C11—H7	119.1
C3—C1—H1	120.0	C10—C11—H7	119.1
C7—C1—H1	120.0	C5—C4—C3	120.8 (4)
O1—C2—C8	122.2 (4)	C5—C4—H4	119.6
O1—C2—C7	118.3 (3)	C3—C4—H4	119.6
C8—C2—C7	119.5 (4)	O3—C6—C7	122.0 (3)
O2—C10—C8	117.8 (4)	O3—C6—C5	116.7 (4)
O2—C10—C11	118.2 (4)	C7—C6—C5	121.4 (4)
C8—C10—C11	124.0 (4)	O4—C9—O3	115.3 (4)
C1—C3—C4	119.9 (4)	O4—C9—C8	127.5 (4)
C1—C3—H3	120.1	O3—C9—C8	117.3 (3)
C4—C3—H3	120.1	C15—C16—C17	120.0 (4)
C15—C14—C13	120.4 (4)	C15—C16—H10	120.0
C15—C14—H12	119.8	C17—C16—H10	120.0
C13—C14—H12	119.8	C4—C5—C6	119.4 (4)
C11—C12—C13	127.0 (4)	C4—C5—H5	120.3
C11—C12—H6	116.5	C6—C5—H5	120.3
C13—C12—H6	116.5	C6—O3—C9	122.9 (3)
C6—C7—C1	118.6 (4)	C16—C17—C18	119.5 (5)
C6—C7—C2	118.2 (3)	C16—C17—H9	120.3
C1—C7—C2	123.2 (4)	C18—C17—H9	120.3
C2—C8—C10	118.1 (4)	C13—C18—C17	121.4 (4)
C2—C8—C9	120.0 (3)	C13—C18—H8	119.3
C10—C8—C9	121.9 (3)	C17—C18—H8	119.3
C16—C15—C14	120.3 (4)	C18—C13—C14	118.5 (4)
C16—C15—H11	119.9	C18—C13—C12	122.1 (4)
C14—C15—H11	119.9	C14—C13—C12	119.3 (4)

### Hydrogen-bond geometry (Å, °)

**Table:**

*D*—H···*A*	*D*—H	H···*A*	*D*···*A*	*D*—H···*A*
C5—H5···O3^i^	0.93	2.57	3.350 (5)	142.
O1—H2···O2	0.99 (7)	1.51 (7)	2.413 (4)	149 (6)

Symmetry codes: (i) −*x*+1, *y*+1/2, −*z*+1/2.
